# Supplementary material for: Novel kind of decagonal ordering in Al74Cr15Fe11
Source: Nat Commun. 2020 Dec 4;11:6209. doi: 10.1038/s41467-020-20077-4 (PMC7718917; doi:10.1038/s41467-020-20077-4)
Supplement: Supplementary file 1 — Supplementary Information [file 41467_2020_20077_MOESM1_ESM.pdf]

Supplementary information for

Novel kind of decagonal ordering in $Al_{74}Cr_{15}Fe_{11}$

Haikun Ma[1], Zhanbing He[1,*], Hua Li[1], Tiantian Zhang[1], Shuang Zhang[2], Chuang Dong[2,3], Walter Steurer[4]

[1]State Key Laboratory for Advanced Metals and Materials, University of Science and Technology Beijing, Beijing 100083, China
[2]School of Materials Science and Engineering, Dalian Jiaotong University, Dalian 116028, China
[3]Key Laboratory for Materials Modification by Laser, Ion and Electron Beams (Dalian University of Technology), Ministry of Education, Dalian 116024, China
[4]Department of Materials, ETH Zurich, 8093 Zurich, Switzerland

*Correspondence should be addressed to: hezhanbing@ustb.edu.cn

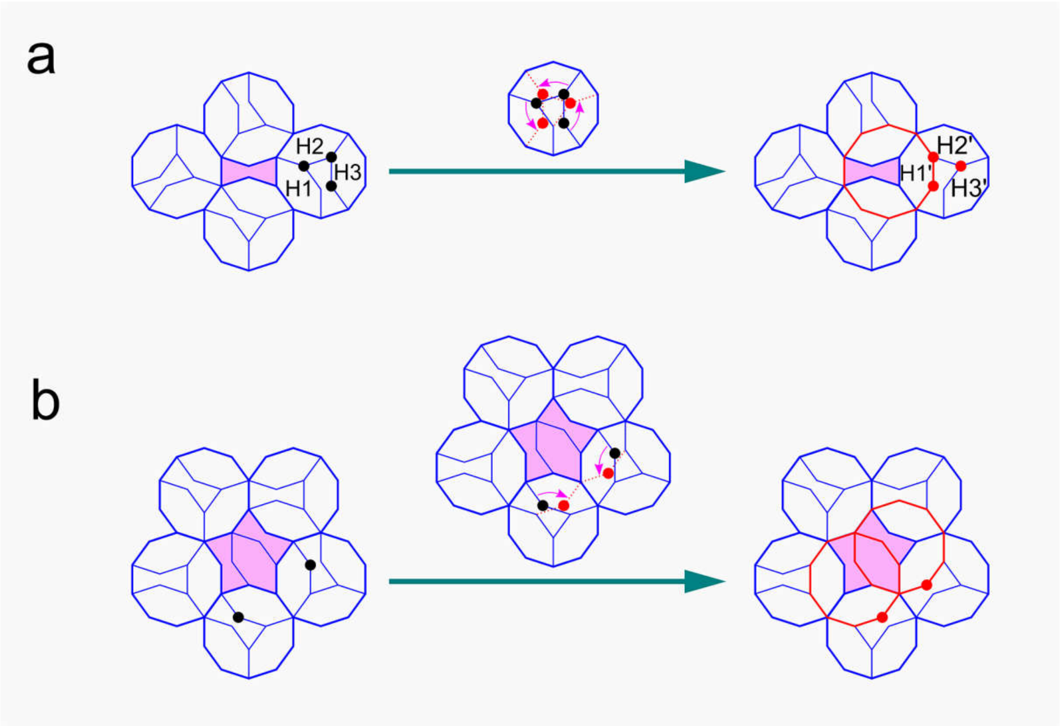

Supplementary Figure 1 Phason flipping to eliminated some defects (namely the purple area). One or two more 2.0 nm decagons are generated in the right column with the disappearance of purple defects in the left column after the action of phason flipping.

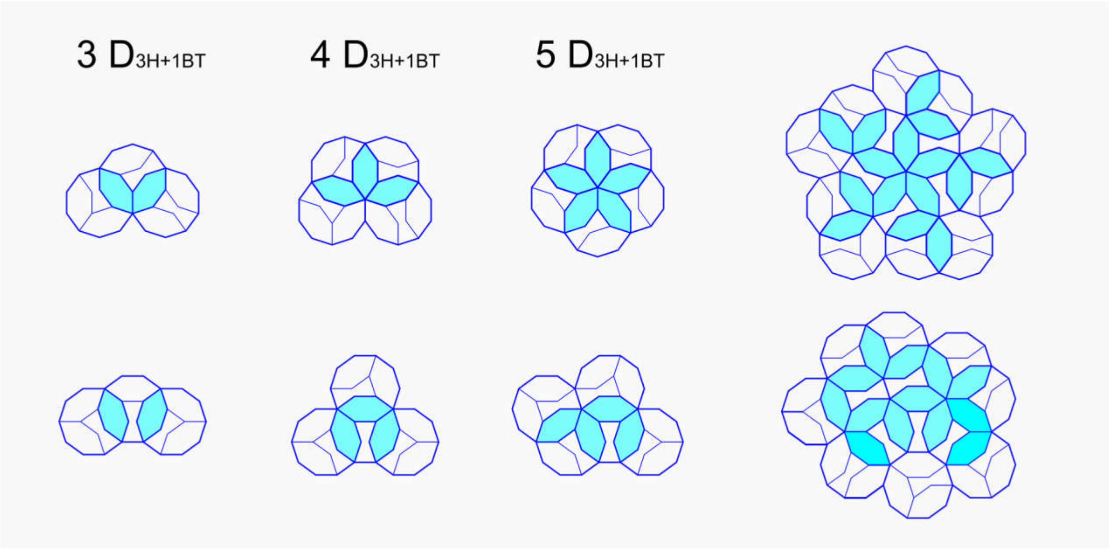

Supplementary Figure 2 The linkages of D_{3H+1BT} tiles without gaps. The connections of three, four, five and more D_{3H+1BT} tiles (from left to right) follow the simple connection rules in Fig. 3b in the paper to cover the whole plane without gaps.
